# Movement Pattern of Scapular Dyskinesis in Symptomatic Overhead Athletes

**DOI:** 10.1038/s41598-017-06779-8

**Published:** 2017-07-26

**Authors:** Tsun-Shun Huang, Jiu-Jenq Lin, Hsiang-Ling Ou, Yu-Ting Chen

**Affiliations:** 1 School and Graduate Institute of Physical Therapy, College of Medicine, National Taiwan University, 100, Taipei, Taiwan; 2 Department of Physical Medicine and Rehabilitation, National Taiwan University Hospital, 100, Taipei, Taiwan; 3 Department of Physical Medicine and Rehabilitation, Taipei Medical University Hospital, Taipei, Taiwan

## Abstract

This study investigated the characteristics of arm elevation via principal component analysis in symptomatic overhead athletes with scapular dyskinesis. One hundred-thirty-four overhead athletes with scapular dyskinesis [24: inferior angle prominence (pattern I); 46: medial border prominence (pattern II), 64: pattern I + II] were evaluated by three-dimensional electromagnetic motion and electromyography to record the scapular kinematics (upward rotation/posterior tipping/exterior rotation) and muscle activation (upper trapezius: UT; middle trapezius: MT; lower trapezius: LT; serratus anterior: SA) during lowering phase of arm elevation. The results showed: (1) for pattern I and II, the first 3 principal component (PCs) explained 41.4% and 42.6% of total variance of movement; (2) the first PCs were correlated with MT, LT activity (r = 0.41~0.61) and upward rotation, posterior tipping (r = −0.59~−0.33) in pattern I, and UT, MT, SA (r = 0.30~0.70) activity in pattern II; (3) contour plots of muscle activity demonstrated that muscle activities varied with dyskinesis patterns. In summary, for the pattern I, the major characteristics are coactivation of MT and LT and corresponding scapular posterior tipping and upward rotation. For the pattern II, the major characteristics are coactivation of UT, MT and SA without corresponding scapular external rotation.

## Introduction

Scapular dyskinesis has been recognized as a non-specific response to a painful condition in the shoulder^1, 2^. Previous research has found that the prevalence of scapular asymmetry in scapular plane elevation and flexion did not differ in asymptomatic and symptomatic participants (71–77% and 71–76%, respectively)^3^. Other studies have demonstrated that various shoulder disorders, including rotator cuff injuries, glenohumeral instability, and labral tears, are associated with scapular dyskinesis, with prevalence rates of 33 to 100%^4–7^. One of the reasons for these non-specific results may be the methods used to assess scapular dyskinesis/asymmetry. With only video/visual observation of dyskinesis, lack of adequate reliability and validity has been reported^3, 8–10^. Recently, the classification of patterns of dyskinesis based on visual observation and palpation has been shown to have sufficient reliability^11^. It is likely that patterns of scapular kinematics are specific to shoulder disorder mechanisms.

Several mechanisms have been proposed to explain shoulder injuries in terms of scapular dyskinesis. Unlike a retracted position of the scapula (subacromial space, average = 10.2 mm), a protracted position of the scapula (subacromial space, average = 8.4 mm) decreases the subacromial space^12^. Excessive protraction during arm elevation may result in decreased subacromial space and impingement^13^. Investigation of scapular kinematics in subjects with and without shoulder injuries identified decreased scapular upward rotation, decreased scapular posterior tilt, and increased scapular elevation in the subjects with shoulder injuries^14–16^. It was proposed that sufficient scapular movements may prevent the greater tuberosity of the humeral head from passing smoothly under the acromion during humeral elevations and lead to impingement syndrome^13, 17–19^. Additionally, excessive activity of the upper trapezius (UT) muscle combined with reduced activity of the lower trapezius (LT) muscle and serratus anterior (SA) muscle have been observed in patients with shoulder impingement^18, 20, 21^. Moreover, lack of control of the functional kinetic chain of the scapula can result in an imbalance of force transmission from the lower extremities and trunk to the upper extremities. This imbalance can also influence skilled shoulder function, as occurs in athletic performance^22–24^. Therefore, scapular movement strategies specific to patterns of dyskinesis should be identified to facilitate the assessment of clinical pathologies and physiological adaptions, as well as that of treatment or rehabilitation effects.

Lack of appropriate scapular kinematics and muscle imbalance may significantly influence movement in overhead athletes such as throwing in baseball or spiking in volleyball because of the cumulative effect of repetitive overhead motions and forces on shoulder complex^13, 19^. Understanding the movement characteristics in patients performing overhead sports may help the treatment and prevention of shoulder disorders in overhead athletes with scapular dyskinesis. The aim of this study was to assess the scapular kinematics and associated muscle activities in overhead athletes with different patterns of scapular dyskinesis to determine whether scapular characteristics were specific to patterns of dyskinesis.

## Results

The scapular dyskinesis pattern distribution in raising and lowering phases were demonstrated as follow: (1) raising phase (pattern I: 1, pattern II: 5, pattern I + II: 1, normal: 127); (2) lowering phase (pattern I: 24, pattern II: 46, pattern I + II: 64). Because most of participants did not show scapular dyskinesis in raising phase, we would only focus on lowering phase in further analyses. As mentioned above, data with pattern I + II dyskinesis were included and analyzed in both the pattern I and the pattern II groups. As a result, the classification of dyskinesis patterns identified 88 participants with inferior angle prominence of the scapula in pattern I and mixed pattern dyskinesis (67 male; age: 23.5 ± 4.3; height: 171.6 ± 8.2; weight: 65.0 ± 9.6) and 110 participants with medial border of the scapula prominence in pattern II and mixed pattern dyskinesis (87 male; age: 23.8 ± 3.7; height: 171.9 ± 7.5; weight: 66.1 ± 10.3) in lowering phase. There were no differences in the demographic data among the 2 patterns. Kinematics and EMG data are presented in Table 1.

**Table 1:** Three-dimensional scapular kinematics and 4-muscle EMG data during lower phase of arm elevation in scapular plane.

Arm elevation	Pattern I (mean ± SD) N = 88					Pattern II (mean ± SD) N = 110				
	>120°	90°~120°	60°~90°	30°~60°	0~30°	>120°	90°~120°	60°~90°	30°~60°	0~30°
UR*		37.5 ± 6.5	28.8 ± 5.2	17.4 ± 5.0	4.8 ± 4.1		38.3 ± 7.1	29.0 ± 5.6	17.5 ± 5.1	4.7 ± 4.1
PT*		8.2 ± 6.9	6.5 ± 5.6	4.2 ± 4.2	1.7 ± 2.7		8.4 ± 7.4	6.6 ± 6.0	4.2 ± 4.3	1.6 ± 2.6
ER*		6.1 ± 10.2	2.6 ± 7.1	0.5 ± 5.0	0 ± 3.6		5.7 ± 10.3	2.7 ± 7.0	0.9 ± 5.4	0.4 ± 4.2
UT	35.5 ± 15.3	31.4 ± 10.1	27.5 ± 8.5	18.2 ± 7.0	8.4 ± 4.6	37.8 ± 17.1	33.3 ± 11.9	29.3 ± 10.9	19.6 ± 9.2	9.1 ± 6.1
MT	23.6 ± 12.1	20.4 ± 8.5	18.0 ± 8.3	13.9 ± 7.1	8.1 ± 4.3	24.7 ± 13.6	20.4 ± 8.6	18.1 ± 8.1	14.1 ± 6.7	8.2 ± 3.9
LT	20.1 ± 13.3	19.0 ± 12.4	18.0 ± 12.8	12.6 ± 8.3	6.7 ± 4.5	21.4 ± 16.0	19.1 ± 13.2	18.1 ± 13.6	12.8 ± 9.9	6.9 ± 4.9
SA	50.0 ± 18.7	36.7 ± 12.5	26.3 ± 10.5	15.2 ± 8.1	8.6 ± 5.8	50.0 ± 18.6	36.3 ± 14.1	25.3 ± 10.7	14.1 ± 7.8	8.1 ± 5.6

Pattern I: Inferior medial angle of the scapula is displaced posteriorly from the posterior thorax, prominent during dynamic observation and palpation; Pattern II: Entire medial border of the scapula is displaced posteriorly from the posterior thorax, prominent during dynamic observation and palpation.

120°, 90°, 60°, 30°: kinematics value at each angle of arm elevation; SD: standard deviation; N: number.

*: 3-dimensional scapular kinematics (degree); UR: upward rotation; PT: posterior tipping; ER: external rotation.

EMG value: percentage of maximal voluntary isometric contraction (% MVIC); UT: upper trapezius; MT: middle trapezius; LT: lower trapezius; SA: serratus anterior.

For pattern I dyskinesis, 3 PCs explained a total of 41.4% of the variance of movement (KMO = 0.579). The first PC (17.5%) was correlated with MT, LT activity (r = 0.41~0.61) and upward rotation, posterior tipping (r = −0.59~−0.33); the second PC (13.4%), with MT activity and scapular external rotation (r = 0.40~0.67); and the third PC (10.5%), with LT activity (r = 0.44~0.57). For pattern II dyskinesis, 3 PCs explained a total of 42.6% of the variance of movement (KMO = 0.632). The first PC (17.0%) was correlated with UT, MT, SA activity (r = 0.30~0.70); the second PC (14.3%), with posterior tipping and LT (r = 0.55~0.72); and the third PC (11.3%), with MT activity and scapular upward rotation and external rotation (r = −0.6~0.47) (Table 2). In summary, for the pattern I, the first PC was described as primary mover/movement characteristic, the second PC was described as secondary dyskinesis characteristic (prominence of medial border) and the third PC was accessory stabilizer. For the pattern II, the first PC was described as stability characteristic, the second PC was described as secondary dyskinesis characteristic (prominence of inferior angle) and the third PC was accessory mover/movement characteristic.

**Table 2:** Principal component analysis, correlation between principal components (PCs) and 3-dimensional scapular kinematics/4-muscle EMG.

Pattern I	PC1	PC2	PC3
upward rotation*	**−0.59~−0.42**	0.24~**0.34**	0.09~**0.46**
posterior tipping*	**−0.50~−0.33**	0.11~**0.37**	−0.13~−0.11
external rotation*	−0.29~−0.13	**0.53~0.59**	−0.20~−0.12
UT	0.26~**0.41**	**−0.34**~−0.22	0.11~0.27
MT	**0.44~0.49**	**0.40~0.67**	0.28~**0.42**
LT	**0.41~0.61**	0.22~**0.32**	**−0.57~−0.44**
SA	0.20~**0.51**	−0.18~0.16	0.26~**0.55**
Pattern II			
upward rotation*	0.07~0.21	0.28~**0.38**	**−0.43** ~ **−0.33**
posterior tipping*	**−0.31**~−0.15	**0.55~0.71**	**−0.30**~−0.09
external rotation*	**−0.30**~−0.09	0.12~**0.33**	**0.31~0.47**
UT	**0.56~0.67**	−0.03~−0.20	−0.01~0.05
MT	**0.57~0.70**	−0.22~0.07	**−0.60~−0.31**
LT	0.24~0.29	**0.59~0.72**	0.27~**0.41**
SA	**0.30~0.50**	0.05~0.23	0.06~**0.36**

Pattern I: Inferior medial angle of the scapula is displaced posteriorly from the posterior thorax, prominent during dynamic observation and palpation; Pattern II: Entire medial border of the scapula is displaced posteriorly from the posterior thorax, prominent during dynamic observation and palpation.

*: 3-dimensional scapular kinematics; UT: upper trapezius; MT: middle trapezius; LT: lower trapezius; SA: serratus anterior.

Bold with or without underline indicates moderate to high correlations.

**Figure 1:**
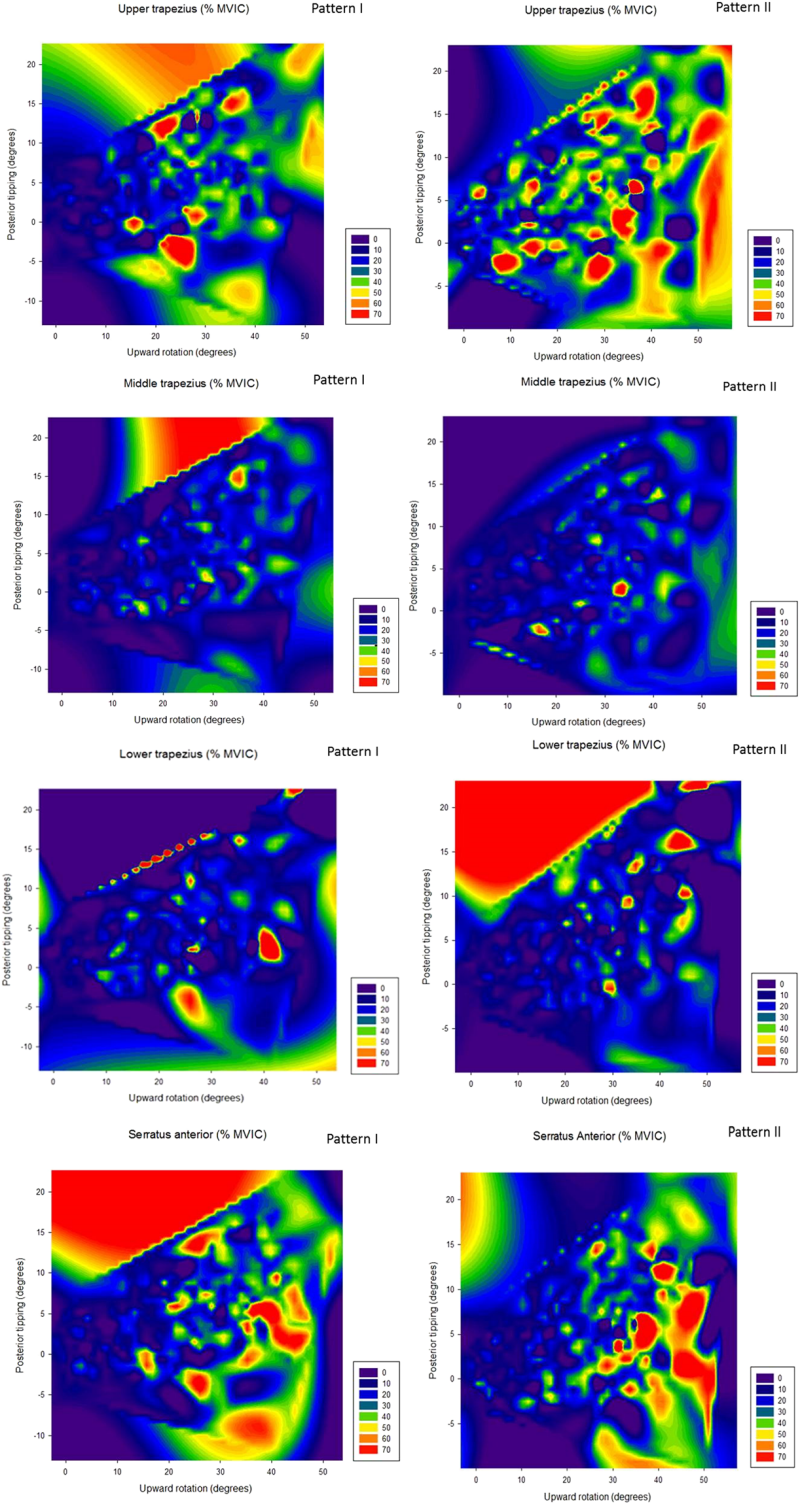
Contour plots of upper/middle/lower trapezius and serratus anterior muscle activity with scapular upward rotation and posterior tipping in X and Y axes, respectively. Pattern I: inferior angle prominence; pattern II: medial border prominence; MVIC: maximal voluntary isometric contraction.

The contour plots of 4-muscle EMG with scapular upward rotation and posterior tipping in the X and Y axes respectively are presented in Fig. 1. Muscle activities were unique to patterns of dyskinesis.

## Discussion

Impaired scapular kinematics and associated muscle activation are thought to result in shoulder disorders such as pain, restricted range of motion, and functional disability. Based on these assumptions, researchers have identified insufficient posterior tipping, external rotation, and upward rotation; decreased serratus anterior and lower trapezius activity; and increased upper trapezius muscle activity in patients with shoulder impingement^16, 18, 20, 21, 25^. Understanding the scapular kinematics and associated muscle activities unique to specific patterns of scapular dyskinesis would be important if the consequences of such alterations are correlated to clinical outcomes and injury mechanisms, especially in overhead athletes. Main findings of our study showed different characteristics in pattern I and pattern II dyskinesis. Main characteristics in pattern I dyskinesis are MT, LT activity and upward rotation, posterior tipping compared to UT, MT, SA activity in pattern II dyskinesis. Our results provide a method of analysis and identify the characteristics of the kinematics and muscle activities that are distinctive to patterns of scapular dyskinesis in subjects performing overhead sports.

Principle component analysis of the scapular kinematics and muscle activities in different patterns of dyskinesis has not been reported previously. Theoretically, scapular kinematics and associated muscle activities during arm movements should share similar variance. However, our findings indicated that the first PC was described as primary mover/movement characteristic (MT, LT activity and upward rotation, posterior tipping) in pattern I versus stability characteristic (only UT, MT, SA activity) for pattern II. External rotation was described as accessory movement characteristics in third PC in pattern II dyskinesis. Thus, change of muscle activation may not be obviously corresponding to scapular movement during arm elevation in pattern II dyskinesis. The results indicated that activation of the scapular muscles plays primary roles as stabilizers and secondary roles as movers of the scapula in patients with pattern II dyskinesis. This may explain why specific muscle activities and theoretically associated scapular kinematics were not found to be highly related in past research^26^. Clinically, this raises the question of whether specific muscle training can change scapular kinematics^27–29^. Further research is needed to validate this assumption. Other studies have reported that excessive activity of the UT combined with reduced activity of the LT and SA was observed during arm elevation in patients with shoulder impingement^18, 20^. Consistent with previous findings, the moderate correlations (0.41–0.70) of the UT, MT, LT, SA shared the major components. This finding implies that these muscles are activated together as a force couple during arm movement.

Scapular muscle activations are unique to each pattern of dyskinesis. MT and LT activities should be considered as characteristics of pattern I dyskinesis while UT, MT, SA activities can be considered as characteristics of pattern II dyskinesis. Additionally, the contour lines and plots expressed the functions of the observed or hypothetical muscle activations on two kinematic variables (upward rotation and posterior tipping). The UT has previously been shown to elevate the scapula and extend the neck, while the MT acts to retract the scapula. Additionally, the SA functions as scapular upward rotator and external rotator^20, 30^. For medial border of scapula prominence (pattern II), UT, MT and SA were demonstrated as principal component without scapular kinematics included in the first PC, which indicated the role of stabilizers were essential in the UT, MT and SA in pattern II dyskinesis. For inferior angle prominence of scapula (pattern I), muscle function of MT, LT and shared variance in the first PC with upward rotation and posterior tipping can be explained to stabilize the axis for scapular upward rotation by MT and to function posterior tipping of the scapula by LT during arm elevation. However, the activation of LT cannot generate adequate scapular posterior tipping against the scapular inferior angle in pattern I dyskinesis. On the other hand, activated SA was not associated with generating scapular external rotation against the scapula medial border in pattern II dyskinesis.

In clinical implication, evaluation of MT and LT muscle activations, as shared the same major variance with scapula kinematics, should be considered to correct inferior angle prominence of pattern I scapula dyskinesis subjects. On the other hand, without shared the variance with scapular kinematics in medial border prominence pattern II dyskinesis subjects, UT, MT and SA activations are likely to play major roles in stabilization of scapula instead of movement of the scapula. Instead of scapular muscle training, correction of medial border prominence dyskinesis may consider other factors, like soft tissue tightness or posture. Validation of this assumption should be further investigated.

The limitations of this study should be noted. First, the KMO values in both dyskinesis patterns are at the borderline of acceptance for PCA analysis. Further studies need to confirm our findings of movement characteristics in different scapular dyskinesis patterns. Second, the kinematics data were measured with humeral elevation of less than 120 degrees to reduce the error of the skin-based method. Validation studies have demonstrated that scapular motion can be accurately estimated for humeral elevation inferior to 120° for elevation in the sagittal plane^31, 32^. Third, movement artifacts and crosstalk cannot be excluded with the use of surface electrodes during dynamic movements. However, the 6-Hz filter and less than 10 kΩ of skin resistance of the EMG data reduced the possible impact^33^. Placing electrode pairs 2 cm apart can minimize the effect of crosstalk from other muscles^34^. Third, results from testing arm elevation in scapular plane may not represent results during functional movement or overhead sport activity. Additionally, participants were generally young and participated in overhead sports in this study. Sedentary or elderly people may have different scapular movement and muscle activation.

## Conclusion

PCA demonstrated that the three PCs accounted for 41% and 43% of variance for pattern I and II dyskinesis, respectively. For the inferior angle prominence dyskinesis, the major characteristics are coactivation of middle and lower trapezius and corresponding scapular posterior tipping and upward rotation. For the medial border prominence dyskinesis, the major characteristics are coactivation of upper/middle trapezius and serratus anterior without corresponding scapular external rotation.

## Methods

This study was a cross-sectional study. All participants performed arm elevation in the scapular plane. The characteristics of the scapular kinematics and associated muscle activities specific to patterns of dyskinesis were identified.

One hundred thirty-four subjects with unilateral shoulder pain and scapular dyskinesis were recruited for this study. The inclusion criteria were (1) age of 20 to 40 years old, (2) with unilateral shoulder pain of less than 5 on a 10-point visual analog scale during arm elevation and (3) demonstration of inferior angle or medial border of scapula prominence during arm elevation. All of the participants performed recreational overhead sports (volleyball: n = 41; baseball: n = 37; badminton: n = 22; weight training: n = 10; swimming: n = 5; basketball: n = 5; other sports: n = 14). Exclusion criteria were a history of stroke, diabetes mellitus, rheumatoid arthritis, rotator cuff tear, surgical stabilization of the shoulder, osteoporosis, or malignancies in the shoulder region. Participants who had pain or disorders of the cervical spine, elbow, wrist, or hand, who had pain radiating from the shoulder to the arm, or who could not elevate their arms to 150 degrees were also excluded. They received written and verbal explanations of the purposes and procedures of the study. All subjects gave written informed consent to the Research Ethics Committee of the National Taiwan University Hospital (approval number 201412043RINA) following a complete explanation of this study, and in accordance with the Declaration of Helsinki.

The Polhemus 3Space FASTRAK system (Polhemus Inc., Colchester, VT, USA), an electromagnetic-based motion analysis system, was used for collecting 3-dimensional kinematic data of the scapula. Karduna *et al*. validated scapular kinematics between skin-based sensor and bone-pinned methods and confirmed that the skin-based method is valid when arm elevation is below 120 degrees^31^. The details of the methodology can be found in a previous paper^26^. Three sensors were placed in locations where the skin motion artifact was minimized (sternum, acromion, distal humerus). Anatomic landmarks (sternal notch, xiphoid process, seventh cervical vertebra, eighth thoracic vertebra, acromioclavicular joint, root of the spine of the scapula, inferior angle of the scapula, lateral epicondyle, and medial epicondyle) were palpated and used for subsequent receiver mounting and landmark digitization. The investigator with 7 years’ clinical experience in musculoskeletal palpation did the anatomic landmarks palpation.

The sEMG assembly comprised pairs of silver chloride circular (recording diameter of 10 mm) surface electrodes (The Ludlow Company LP, Chocopee, MA) with an interelectrode (center- to center) distance of 20 mm, and a Grass AC/DC amplifier (Model 15A12, Astro-Med Inc. RI, USA) with a gain of 1000, a common mode rejection ratio of 86 dB at 60 Hz, and a bandwidth (−3 dB) of 10 to 500 Hz. The sEMG data were collected at 1,000 Hz/channel using a 16-bit analog to digital converter (Model MP 150, Biopac systems Inc., CA, USA). Surface EMG electrodes were placed on the upper trapezius (UT, midway between the acromion and C7), middle trapezius (MT, midway between the root of the spine of the scapula and T3), lower trapezius (LT, on the line between the spine of the scapula and T7) and serratus anterior (SA, anterior to the latissimus dorsi and posterior to the pectoralis major) of the involved shoulder. The referenced electrode was placed on the ipsilateral clavicle. Maximal voluntary isometric contraction (MVIC) was tested and used to normalize the sEMG data during the task (for the UT, resisted shoulder flexion of 90 degrees; for the MT, resisted horizontal abduction while lying prone with the arm abducted to 90 degrees; for the LT, resisted arm elevation while lying prone with the arm abducted in line with muscle fibers; and for the SA, resisted arm elevation of 135 degrees). The MVICs were collected for 5 seconds in each of 3 trials, with 1 minute of rest separating trials.

Visual combined palpation was used to classify scapular position and movement patterns (single patterns or mixed patterns) in both the raising and the lowering phases, modified by Kibler’s method^8, 11^. The inter-rater reliability of the classification test was moderate to substantial (κ coefficients = 0.49 and 0.57/0.64 in the raising and lowering phases, respectively)^11^. Two single patterns with inferior angle of the scapula prominence (pattern I) and medial border of the scapula prominence (pattern II) and the mixed pattern with combination of the two single patterns were selected in this study.

Kinematics and sEMG data were collected during arm elevation in the scapular plane. Participants were asked to elevate the arms that the dumbbells in each hand weighed 2.3 kg (5 lb) or 1.4 kg (3 lb), depending on each participant’s ability to elevate the arm. In general, male participants lifted 5 lb and female participants used 3 lb of resistance. Raw kinematic data were low-pass filtered at a 6-Hz cutoff frequency and converted into anatomically defined rotations. In general, we followed the ISB guidelines for constructing a shoulder joint coordinate system^35^. Scapular orientation relative to the thorax was described using a Euler angle sequence of rotation about Z_s_ (protraction/retraction), rotation about Y’_s_ (downward/upward rotation), and rotation about X’_s_ (posterior/anterior tipping). Full bandwidth sEMG data captured by the data acquisition software (AcqKnowledge, Biopac systems Inc., CA, USA) were reduced using a root mean square (RMS) algorithm to produce sEMG envelopes with an effective sampling rate of 50 samples. Then the data were normalized to the MVIC trials. The EMG data of each muscle were the average of the middle 3 trials. The mean sEMG amplitude of each muscle, reported as a percentage of MVIC, was used to assess the activity of the muscle. For the analysis, 4 levels at 30°, 60°, 90°, and 120° of arm elevation for kinematics and 5 levels during 0–30°, 30–60°, 60–90°, 90–120° and above 120° of arm elevation for EMG data were used.

The Statistical Package for the Social Sciences (SPSS) 17.0 was used for data analysis. Descriptive statistics of kinematics and EMG data were calculated. Data of participants with pattern I + II dyskinesis were included and analyzed in both the pattern I and the pattern II groups. To identify the characteristics of each dyskinesis pattern, principal component analysis (PCA) was performed on all data (data input matrix of 4 levels at 30°, 60°, 90°, and 120° of arm elevation for 3-dimensional scapular kinematics and 5 levels during 0–30°, 30–60°, 60–90°, 90–120° and above 120° of arm elevation for 4 muscles EMG) for each dyskinesis pattern with varimax transformation. This analysis yielded the amount of variance explained by each principal component, and also the correlations between principal components (PCs) and the 3-dimensional scapular kinematics and 4 muscles EMG. The Kaiser-Meyer-Olkin (KMO) Measure of sampling adequacy was used to test the appropriateness of using PCA^36^. Additionally, the characteristics of movements of the dyskinesis patterns were demonstrated by contour plots of 4-muscle EMG with scapular upward rotation and posterior tipping in the X and Y axes, respectively.
